# Diagnostic accuracy of the loop-mediated isothermal amplification assay for extrapulmonary tuberculosis: A meta-analysis

**DOI:** 10.1371/journal.pone.0199290

**Published:** 2018-06-26

**Authors:** Guocan Yu, Yanqin Shen, Fangming Zhong, Bo Ye, Jun Yang, Gang Chen

**Affiliations:** 1 Department of Thoracic Surgery, Hangzhou Red Cross Hospital, Hangzhou, Zhejiang, China; 2 Department of Tuberculosis, Hangzhou Red Cross Hospital, Hangzhou, Zhejiang, China; Food and Drug Administration, UNITED STATES

## Abstract

**Background:**

Loop-mediated isothermal amplification (LAMP) is used to detect pulmonary tuberculosis (PTB); however, the diagnostic accuracy of the LAMP assay for extrapulmonary tuberculosis (EPTB) is unclear. We performed a meta-analysis to evaluate the performance of LAMP in the detection of EPTB.

**Methods:**

We searched PubMed, EMBASE, the Cochrane Library, China National Knowledge Infrastructure (CNKI), and the Wanfang database for studies published before Sep 16, 2017. We reviewed studies and compared the performance of LAMP with that of a composite reference standard (CRS) and culture for clinically suspected EPTB. We used a bivariate random-effects model to perform meta-analyses and used meta-regression and subgroup analysis to analyze sources of heterogeneity.

**Results:**

Fourteen articles including 24 independent studies (16 compared LAMP to CRS, 8 to culture) of EPTB were identified. LAMP showed a pooled sensitivity of 77% (95% confidence interval (CI) 68–85), specificity of 99% (95% CI 96–100), and area under SROC curves (AUC) of 0.96 (95% CI 0.94–0.97) against CRS. It showed a pooled sensitivity of 93% (95% CI 88–96), specificity of 77% (95% CI 64–86), and AUC of 0.94 (95% CI 0.92–0.96) against culture. The pooled sensitivity, specificity, and AUC of MPB64 LAMP were 86% (95% CI 86–86), 100% (95% CI 100–100), and 0.97 (95% CI 0.95–0.98), respectively, and those of IS6110 LAMP were 75% (95% CI 64–84), 99% (95% CI 90–100), and 0.91 (95% CI 0.88–0.93), respectively, compared with CRS.

**Conclusions:**

These results suggest good diagnostic efficacy of LAMP in the detection of EPTB. Additionally, the diagnostic efficacy of MPB64 LAMP was superior to that of IS6110 LAMP.

## Introduction

Tuberculosis (TB) is an infectious disease caused by *Mycobacterium tuberculosis* (MTB) and is one of the most serious challenges to public health [1]. The most common site of tuberculosis infection is the lung, but bacteria can also spread to extrapulmonary sites, causing extrapulmonary tuberculosis (EPTB). EPTB accounts for approximately 22% of total TB cases [2]. Diagnosis of EPTB is very challenging, because specimens of EPTB are not as easy to obtain by noninvasive methods as are sputum samples. Invasive procedures requiring special expertise are often required to obtain specimens such as cerebrospinal fluid and pleural effusion. Additionally, culture of EPTB specimens has low sensitivity. Biopsy along with histopathological examination and culture is required to diagnose EPTB.

There are many methods for diagnosis of tuberculosis. PCR tests require an expensive thermal cycler to amplify DNA fragments in multiple temperature-dependent steps. Therefore, some PCR assays, such as Xpert MTB/RIF, are very costly, which is an obstacle to application in low-income areas. Loop-mediated isothermal amplification (LAMP) is an isothermal DNA method that relies on two or three sets of primers to amplify minute quantities of DNA within a shorter period of time. Compared with other nucleic acid amplification tests, LAMP is very economical. This is a new assay with high accuracy for pulmonary TB detection [3], but there are no systematic studies assessing its diagnostic accuracy for EPTB.

For this purpose, we performed a meta-analysis to reveal the diagnostic test accuracy of the LAMP assay for EPTB using data from previous studies of the LAMP assay compared with that of a composite reference standard (CRS) and culture reference in the detection of EPTB. We analyzed the pooled sensitivity and specificity of this assay against different references. Moreover, the diagnostic efficiency of the test according to different target genes, types of samples, incubation times, condition of samples, and types of LAMP were evaluated by subgroup analysis.

## Methods

We followed the standard guidelines to perform this meta-analysis [4–6].

### Data sources and search strategy

We searched PubMed, EMBASE, the Cochrane Library, China National Knowledge Infrastructure (CNKI), and the Wanfang database for studies evaluating LAMP accuracy in TB published before Sep 16, 2017. The search formula ((“Loop-Mediated Isothermal Amplification” OR LAMP) AND (“Tuberculosis”[Mesh] OR “Tuberculoses” OR “Kochs Disease” OR “Disease, Kochs” OR “Koch's Disease” OR “Disease, Koch's” OR “Koch Disease” OR TB)) was used for PubMed without any language restrictions. The search formulas for EMBASE, the Cochrane Library, CNKI, and the Wanfang database were similar to the PubMed search formula. The search strategies for each database were shown in the S1 File. References of included articles and published reviews were also reviewed for possible candidate studies. We extracted data including author, year, country, true positive (TP), false positive (FP), false negative (FN), true negative (TN) values for the assay, reference standard, target gene, and specimen type, as well as other parameters.

### Inclusion criteria

We included full text original studies assessing the diagnostic accuracy of the LAMP assay for EPTB using extrapulmonary site specimens. Reference standards were defined in the studies and were appropriate. Articles directly provided TP, FP, FN, and TN values for the assay, or included the data necessary to calculate these measures. Case reports, studies of fewer than 10 samples, abstracts, and conference reports without full articles were excluded.

### Reference standard

A composite reference standard (CRS) or mycobacterial culture was defined as the reference standard in the studies. Clinical manifestation, biochemical testing results, smears, histopathology, other nucleic acid amplification tests (NAATs), culture, or a response to anti-tuberculosis treatment constituted the reference standards in the CRS.

### Literature screening and selection

Two investigators independently assessed candidate articles by reviewing titles and abstracts, then full text for inclusion. Discrepancies between the two decisions were resolved by discussion with a third investigator.

### Data extraction

The same two investigators independently extracted the necessary information from each of the included articles. We then cross-checked the information obtained by the two investigators. Discrepancies between the two data sets were settled by discussion with a third investigator, just as in the literature selection phase. Data from studies against two different reference standards or target genes were treated separately.

### Assessment of study quality

According to the two reference standards (CRS and culture), the two investigators independently divided studies into two groups and used a revised tool for Quality Assessment of Diagnostic Accuracy Studies (QUADAS-2) to assess study quality separately [7]. Publication bias was not assessed, because these methods are not applicable for studies of diagnostic accuracy [8].

### Data synthesis and statistical analysis

We first obtained the numbers of TP, FP, FN, and TN in each included study, and then calculated the estimated pooled sensitivity and specificity of LAMP associated with 95% CI against CRS or culture using bivariate random-effects models. Forest plots of the sensitivity and specificity as well as summary receiver operating characteristic (SROC) curves were generated for each study. The area under SROC curves (AUC) was likewise calculated. The I^2^ statistics were calculated to assess the heterogeneity between studies compared with a standard reference. A value of 0% indicated no observed heterogeneity, and values greater than 50% were considered substantially heterogeneous [9, 10]. We explored targeted genes, types of samples, incubation time, condition of samples and types of LAMP as potential sources of heterogeneity using subgroup and meta-regression analyses. At least four available studies were needed to carry out the meta-analysis for a predefined variable type. Data from studies against CRS and culture were analyzed separately. Stata version 14.0 (Stata Corp, College Station, TX, USA) with the MIDAS command packages was used to analyze the results.

### Imperfect reference standard

Imperfect reference standards may lead to misclassification of samples in diagnostic validity studies [11, 12]. For the paucibacillary nature of EPTB, culture is an imperfect reference standard and leads to an underestimation of the true specificity of LAMP. A CRS is a composite standard that comprises the results of several tests; however, a CRS itself may have reduced specificity that could result in apparent FN LAMP results, also leading to an underestimation of the true sensitivity of LAMP [13, 14]. Therefore, a study comparing LAMP with both culture and CRS might provide a more credible range for sensitivity and specificity.

## Results

### Identification of studies and study characteristics

**Fig 1:**
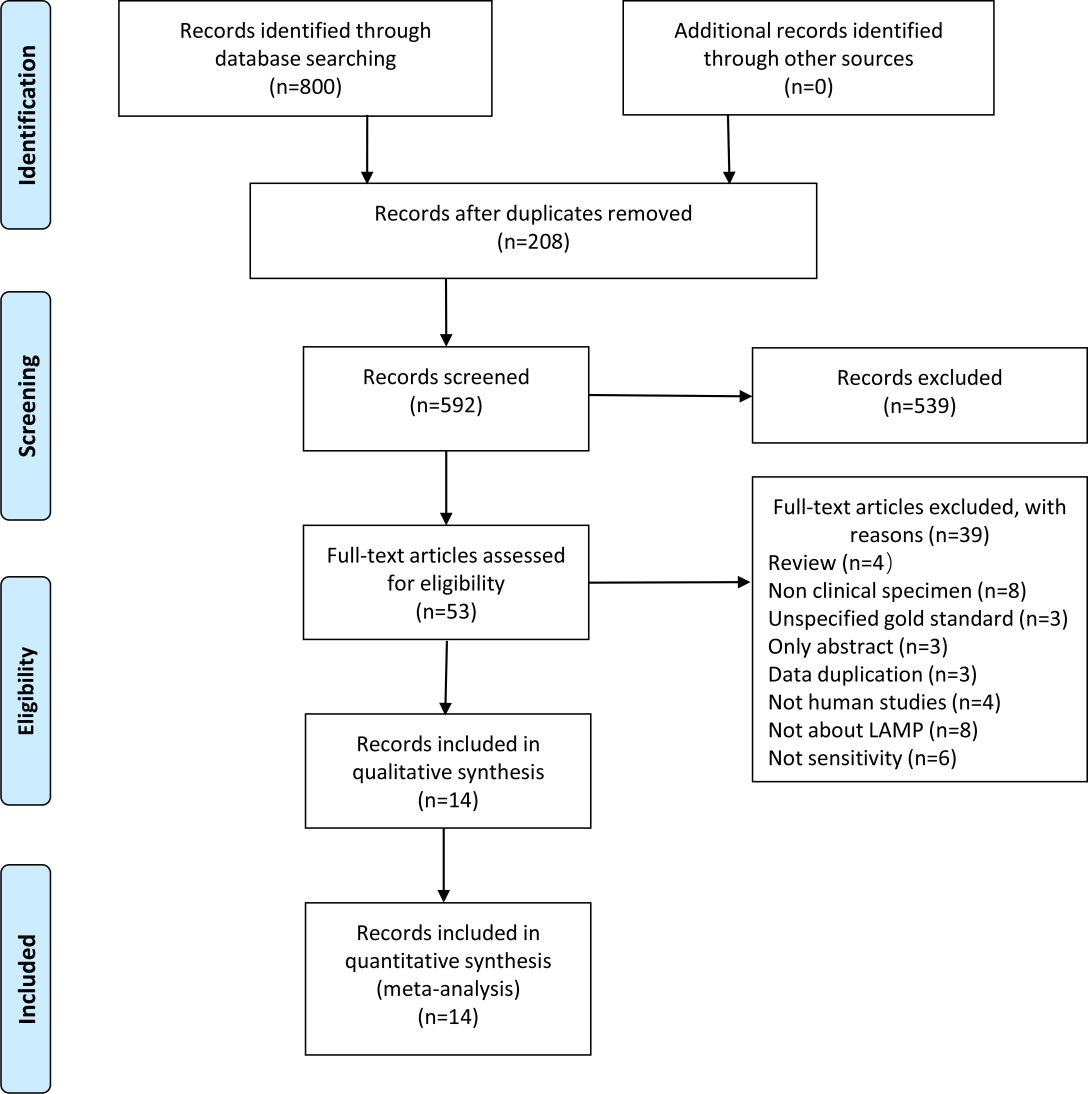
Literature retrieval flow chart. Searches of PubMed, the Cochrane Library, Embase, Wanfang database and CNKI returned 264, 11, 328, 98, and 99 articles, respectively.

Eight hundred candidate articles were identified by searching the relevant databases using our search strategy, and 14 qualified articles were included according to the inclusion criteria (Fig 1, S2 File) [15–28]. The number of specimens evaluated in each article ranged from 27 to 315 with a median of 118. Twelve articles were written in English, and 2 in Chinese. All studies were conducted in countries with high tuberculosis burdens (India and China). We excluded two studies [29, 30] that had the same data as other included studies [15, 25], and one study [31] whose data was part of another study [28].

When an article reported the use of two different standards or target genes in the same study, we considered the article to include two independent studies. In accordance with this principle, 24 independent studies were included: 16 compared LAMP with CRS and 8 with culture (Table 1, S1 Table). The characteristics of the LAMP test used in the 14 articles are also shown in Table 1. The most common target genes were IS6110 and MPB64, used in 11 and 5 studies, respectively. Two studies [16, 28] did not define the target gene. The incubation temperatures of all experiments were similar, at approximately 65°C. The specimens included cerebrospinal fluid (CSF), pleural effusion, synovial fluid, pus, fine needle aspiration (FNA) of lymph glands, and others. Eight articles used only one type of specimen (e.g., only CSF) [20–22, 24–28]. The other studies used multiple types of specimens [15–19, 23]. Only one study provided HIV infection status [26]. Only two studies provided median or mean age. The CRS criteria used in the articles included the results of culture.

**Table 1:** Characteristics of the included studies.

Study	Country	TP(n)	FP(n)	FN(n)	TN(n)	Sensitivity(%)	Specificity(%)	Reference	Gene	Incubation time(min)	Incubation temperature(°C)	Sample condition	LAMP assay	Sample Type
BinFeng Yang_a_ 2011	China	18	0	54	24	25%	100%	CRS	IS1081	60	65	Unknown	Loopamp MTBC	PE
BinFeng Yang_b_ 2011	China	35	4	37	20	49%	83%	CRS	IS1081	90	65	Unknown	Loopamp MTBC	PE
Nagdev, K. J. 2011	India	15	2	2	8	88%	80%	CRS	IS6110	60	63	Frozen	Loopamp MTBC	CSF
Shihui Zhang 2012	China	31	6	2	19	94%	76%	Culture	Unknown	60	65	Fresh	Unclear	Urine
Kumar, P. 2014	India	22	23	0	32	100%	58%	Culture	esat6	35	65	Unknown	In-house	Extrapulmonary samples
Yang Liu 2015	China	44	2	14	30	76%	94%	CRS	hspX	60	63	Fresh	Loopamp MTBC	PE
Joon, D._a_ 2015	India	28	23	2	262	93%	92%	Culture	sdaA	60	65	Frozen	In-house	Extrapulmonary samples
Joon, D._b_ 2015	India	49	2	4	260	92%	99%	CRS	sdaA	60	65	Frozen	In-house	Extrapulmonary samples
Balne, P. K. 2015	India	25	0	8	20	76%	100%	CRS	MPB64	60	65	Frozen	In-house	Vitreous and aqueous humor
Sharma, M._a_ 2016	India	96	0	24	50	80%	100%	CRS	IS6110	45	63	Frozen	In-house	FNA
Sharma, M._b_ 2016	India	103	0	17	50	86%	100%	CRS	MPB64	45	63	Frozen	In-house	FNA
Sethi, S._a_ 2016	India	5	57	2	236	71%	81%	Culture	IS6110	60	64	Fresh	In-house	Endometrial biopsy samples
Sethi, S._b_ 2016	India	45	17	23	215	66%	93%	CRS	IS6110	60	64	Fresh	In-house	Endometrial biopsy samples
Modi, M._a_ 2016	India	46	78	4	122	92%	61%	Culture	IS6110	45	63	Frozen	In-house	CSF
Modi, M._b_ 2016	India	48	82	2	118	96%	59%	Culture	MPB64	45	63	Frozen	In-house	CSF
Modi, M._c_ 2016	India	124	0	26	100	83%	100%	CRS	IS6110	45	63	Frozen	In-house	CSF
Modi, M._d_ 2016	India	130	0	20	100	87%	100%	CRS	MPB64	45	63	Frozen	In-house	CSF
Wenwen Sun_a_ 2017	China	74	2	98	26	43%	93%	CRS	IS6110	40	67	Frozen	Loopamp MTBC	CSF
Wenwen Sun_b_ 2017	China	20	56	2	122	91%	69%	Culture	IS6110	40	67	Frozen	Loopamp MTBC	CSF
Sharma, K._a_ 2017	India	75	0	15	50	83%	100%	CRS	IS6110	45	63	Frozen	In-house	Synovial fluid and pus
Sharma, K._b_ 2017	India	79	0	11	50	88%	100%	CRS	MPB64	45	63	Frozen	In-house	Synovial fluid and pus
Joon, D._a_ 2017	India	43	0	10	262	81%	100%	CRS	IS6110	90	63	Frozen	In-house	Extrapulmonary samples
Joon, D._b_ 2017	India	26	17	4	268	87%	94%	Culture	IS6110	90	63	Frozen	In-house	Extrapulmonary samples
Ghosh, P. K. 2017	India	22	1	1	21	96%	95%	CRS	Unknown	60	65	Frozen	In-house	Extrapulmonary samples

CRS: Composite reference standard. PE: Pleural effusion. FNA: Fine needle aspiration. CSF: Cerebrospinal fluid. Extrapulmonary samples included PE, CSF, and others.

### Study quality

**Fig 2:**
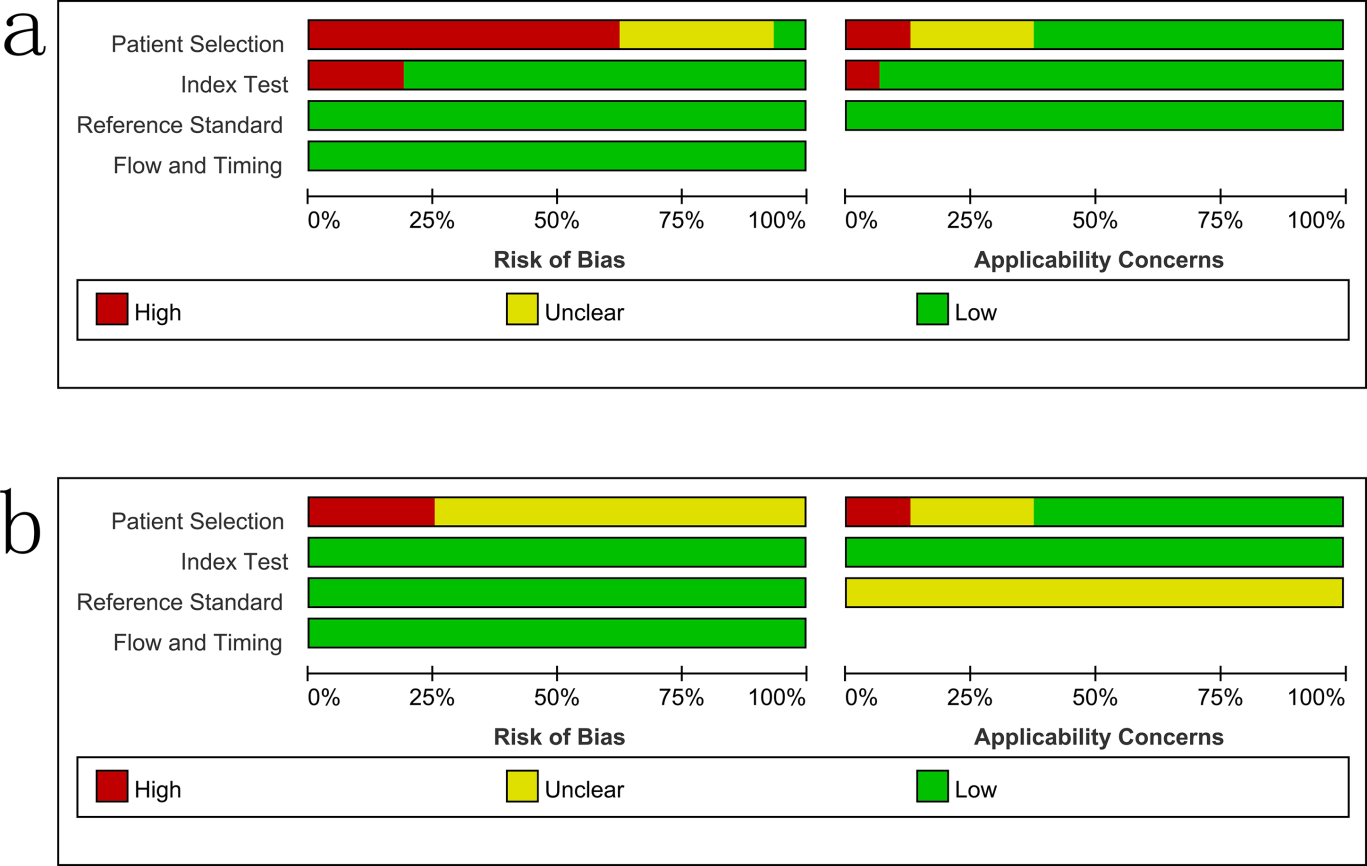
Methodological quality graphs (risk of bias and applicability concerns) as percentages across the included studies. a: composite reference standard. b: culture reference standard.

The overall methodological quality of the included studies using a CRS and culture is summarized in Fig 2. Only four studies used a case–control design [15, 21, 26, 27].

### Diagnostic accuracy of the LAMP assay for EPTB detection

**Fig 3:**
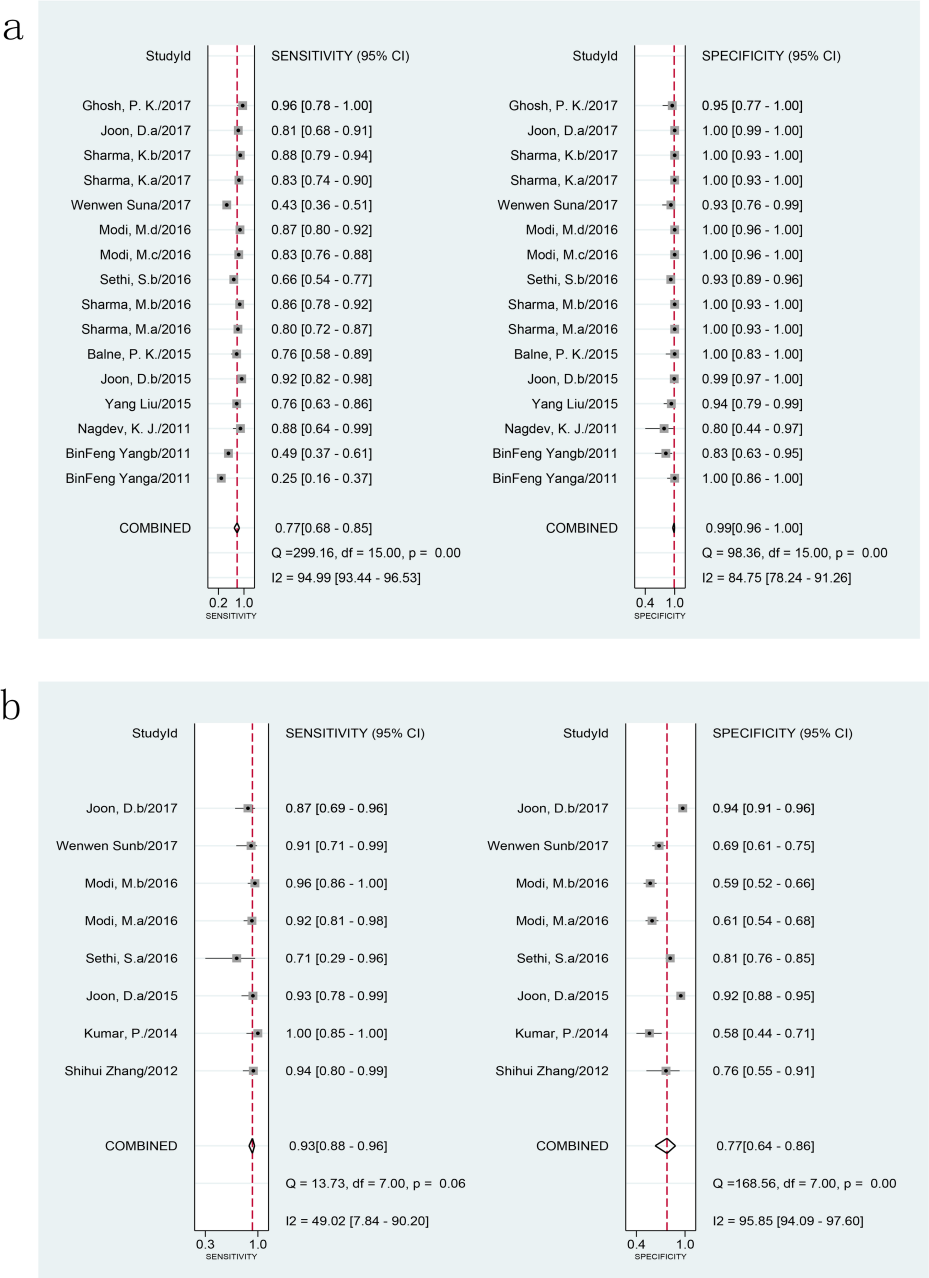
Forest plot of LAMP sensitivity and specificity for tuberculosis detection in EPTB. a: composite reference standard. b: culture reference standard. The squares represent the sensitivity and specificity of a study, and the black line their confidence intervals. The diamonds represent the pooled sensitivity and specificity and their confidence intervals. LAMP: loop-mediated isothermal amplification; EPTB: extrapulmonary tuberculosis.

When compared to a CRS using 2001 samples in 16 studies, the combined sensitivity and specificity of the LAMP assay for EPTB were 77% (95% CI 68–85) and 99% (95% CI 96–100), respectively (Fig 3A). The I^2^ statistical values were 95% for sensitivity and 85% for specificity, suggesting significant heterogeneity in diagnostic validity between the studies. When compared to a culture reference standard (8 studies, 1515 samples), the combined sensitivity of LAMP was 93% (95% CI 88–96) with I^2^ = 49% and the specificity was 77% (95% CI 64–86) with I^2^ = 96% for 1515 specimens in 8 studies (Fig 3B). The heterogeneity of the sensitivity was acceptable; however, the heterogeneity of the specificity was significant. The AUC of SROC was 0.96 (95% CI 0.94–0.97) and 0.94 (95% CI 0.92–0.96) versus vs CRS and culture, respectively, suggesting very good overall diagnostic validity.

We explored the heterogeneity between the studies using hierarchical analysis on predefined subgroups of target genes, sample types, incubation time, condition of samples and types of LAMP used in the assay.

**Fig 4:**
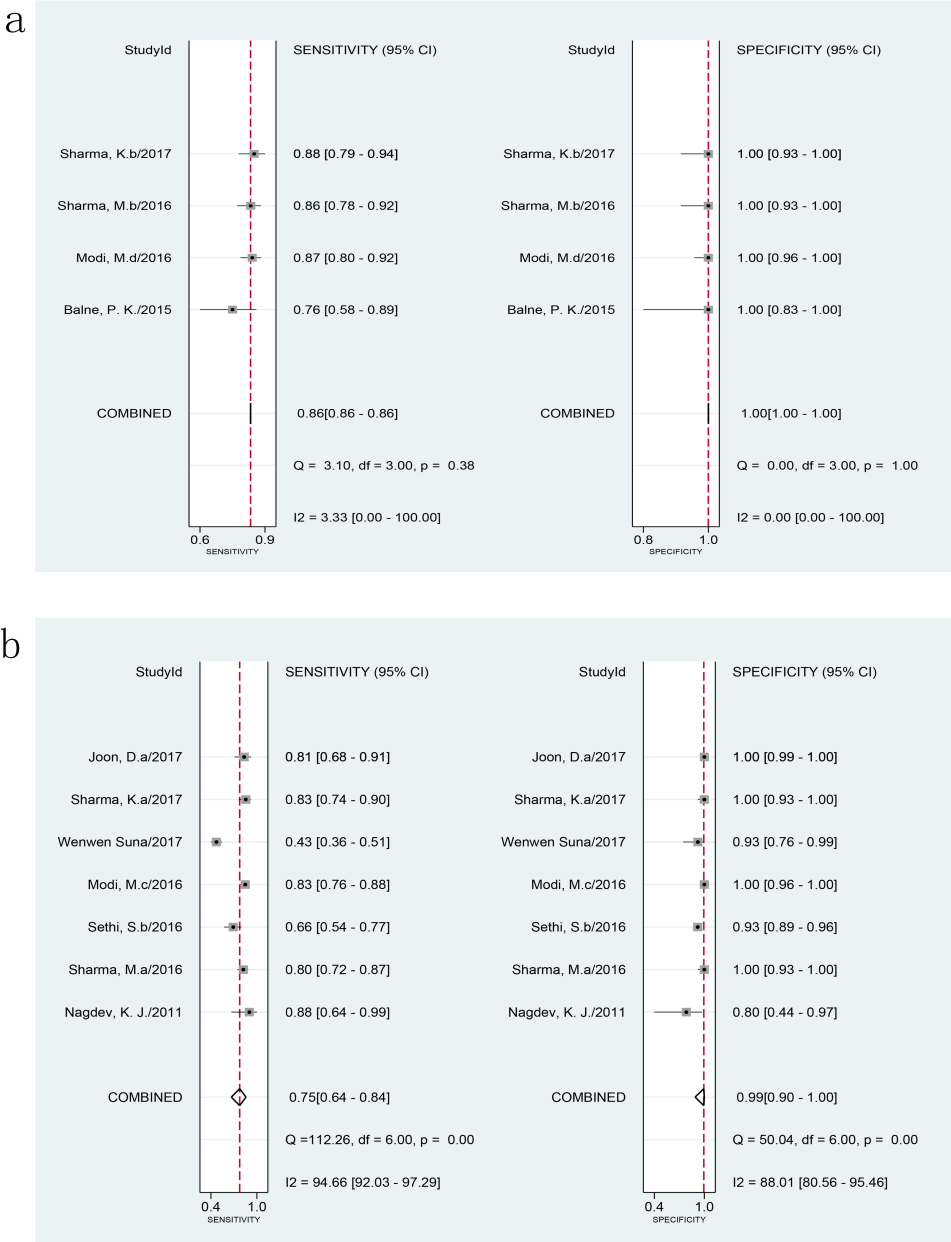
Forest plot of LAMP sensitivity and specificity for tuberculosis detection using different target genes against a composite reference standard. a: MPB64. b: IS6110. The squares represent the sensitivity and specificity of one study, and the black line their confidence intervals. The diamonds represent the pooled sensitivity and specificity and their confidence intervals. LAMP: loop-mediated isothermal amplification.

The pooled sensitivity and specificity of the MPB64 LAMP assay (613 samples) vs. CRS were 86% (95% CI 86–86) with I^2^ = 3.33% and 100% (95% 100–100) with I^2^ = 0, respectively (Fig 4A). There was no heterogeneity in diagnostic validity between studies of MPB64 LAMP. The AUC of SROC was 0.97 (95% CI 0.95–0.98) for MPB64 LAMP vs CRS, suggesting very high efficiency. One study used MPB64 as the target gene in the LAMP assay compared with culture, but further analysis could not be carried out.

When using IS6110 as the target gene, the pooled sensitivity and specificity of IS6110 LAMP compared with CRS were 75% (95% CI 64–84) and 99% (95% CI 90–100), respectively (Fig 4B). I^2^ statistical values were 95% and 88% for sensitivity and specificity, respectively, of IS6110 LAMP. The P values of meta-regression for sensitivity and specificity of the IS6110 LAMP assay against a non-IS6110 LAMP assay in comparison to CRS were 0.16 and 0.25, respectively, suggesting that this target gene was not a source of heterogeneity in the LAMP assay. Therefore, combining different studies to assess the diagnostic performance of the LAMP assay did not significantly skew the results. Compared with culture, the pooled sensitivity of IS6110 LAMP was 89% (95% CI 81–94), and specificity was 79% (95% CI 62–90). The I^2^ statistical values of IS6110 LAMP were 42% and 97% for sensitivity and specificity, respectively. Heterogeneity of sensitivity among the studies was moderate. The pooled sensitivity of MPB64 LAMP was significantly higher than that of IS6110 LAMP (P<0.05); however, the difference between the specificities was not statistically significant (P>0.05), and the AUC of MPB64 LAMP was higher than that of IS6110 LAMP when assessed against CRS. Data of other target genes were too limited to analyze.

**Fig 5:**
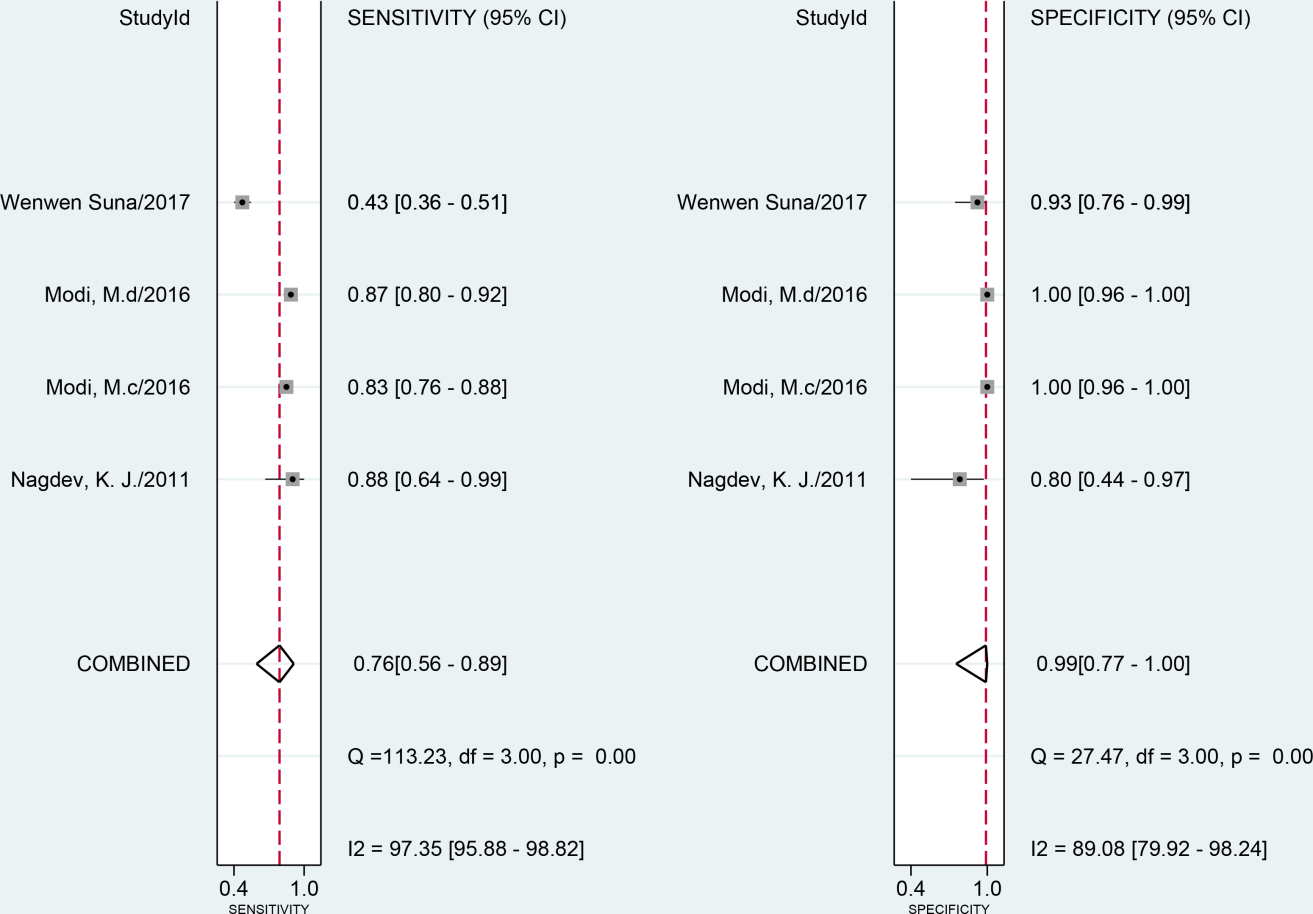
Forest plot of LAMP sensitivity and specificity using cerebrospinal fluid samples against a composite reference standard. The squares represent the sensitivity and specificity of one study, and the black line their confidence intervals. The diamonds represent the pooled sensitivity and specificity and their confidence intervals. LAMP: loop-mediated isothermal amplification.

Four studies assessed LAMP in CSF samples in comparison to a CRS. Pooled sensitivity was 76% (95% CI 56–89, I^2^ = 97%), and pooled specificity was 99% (95% CI 77–100, I^2^ = 89%) (Fig 5). The P-values of meta-regression for sensitivity and specificity were 0.33 and 0.29, respectively. The AUC of SROC was 0.99 (95% CI 0.77–1.00) for CSF samples vs CRS. Sensitivity and specificity of LAMP for pleural effusion against CRS ranged from 25% to 75.8% and 83.3% to 100%, respectively. For fine needle aspiration of lymph nodes, synovial fluid, and pus, sensitivity of this assay was 80%, 85.3%, and 83.3%, respectively. Sensitivity was 87.7% for IS6110 and MPB64 vs CRS, and specificity was consistent at 100%. However, data were too limited to perform meta-analysis.

When LAMP was compared to CRS, meta-regression showed that the type of LAMP was the source of heterogeneity (P < 0.05) rather than incubation time and sample condition (meta-regression P > 0.05). The pooled sensitivity and specificity of Loopamp MTBC (Eiken Chemical, Co., Tokyo, Japan) and in-house assays were 54% (95% CI 40–69) 93% (95% CI 84–100) and 84% (95% CI 79–90), 100% (95% CI 99–100), respectively. The differences between sensitivity and specificity were statistically significant (P < 0.01 and P = 0.03, respectively). However, heterogeneity in the subgroup was still relatively significant, and the results should be treated with caution.

## Discussion

Timely and accurate diagnosis of tuberculosis is very important for effective management of the disease and prevention of infection in the community, particularly in areas with high burdens tuberculosis. Conventional diagnostic methods, such as smears and culture, are time-consuming and not very sensitive.

LAMP is an innovative point-of-care diagnostic technique with increased specificity, speed, and low cost [32]. It can provide results within 1 or 1.5 hours. Several studies have evaluated the diagnostic validity of the test for pulmonary TB [33, 34]. A systematic review and meta-analysis reported by Nagai et al. showed summary estimates of sensitivity at 89.6% (95% CI 85.6–92.6%) and specificity at 94.0% (95% CI 91.0–96.1%) and a diagnostic odds ratio (DOR) of 145 (95% CI 93–226) [35]. However, there has been no reported systematic review and meta-analysis evaluating the diagnostic accuracy of LAMP for EPTB. Ours is the first study for this purpose.

In this meta-analysis, we reviewed the diagnostic efficiency of the LAMP assay for EPTB compared with that of a CRS or culture reference. Based on AUC, the diagnostic performance of the LAMP assay was very good for EPTB, regardless of the reference standard used. However, this test was less effective than PCR assays such as Xpert MTB/RIF [36, 37]. We found that LAMP had very high pooled specificity (99% 95% CI 96–100) but more moderate pooled sensitivity (77% 95% CI 68–85) for the diagnosis of EPTB vs. CRS. As expected, when culture was used as the reference standard, the pooled sensitivity for the diagnosis of EPTB was improved to 93% (95% CI 88–96), and pooled specificity decreased to 77% (95% CI 64–86). However, there was obvious heterogeneity among the studies, and the results should be interpreted carefully.

For the detection of the MTB genome, several factors play important roles in standardizing a sensitive and specific LAMP assay. The target gene is an important factor, and a variety of target genes can be used in NAATs. In LAMP, the commonly used target genes are IS6110, MPB64, and IS1081, among others. Through this meta-analysis, we found that diagnostic efficacy was different when using different target genes. The IS6110 gene has been the favored target gene in studies using the LAMP assay, as multiple copies are present in the MTB genome [38, 39]. However, in this study, it was not the most efficient target gene for diagnosis of EPTB in areas with high burdens of tuberculosis. We observed that the pooled sensitivity and specificity of MPB64 LAMP were significantly higher than those of non-MPB64 LAMP (P = 0.03 and P = 0.00, respectively) when compared to CRS.

Heterogeneity between the studies using MPB64 LAMP was not significant. The pooled sensitivity and specificity of IS6110 LAMP compared with those of non-IS6110 LAMP were not significantly different vs. CRS. However, heterogeneity between IS6110 LAMP studies was very significant. The pooled sensitivity and AUC of MPB64 LAMP were higher than those of IS6110 LAMP against CRS. This result was consistent with those of studies using other NAATs [40, 41]. Included studies using culture as the reference standard were limited, and the difference in pooled sensitivity and specificity for the two target genes could not be analyzed.

We considered that LAMP accuracy for tuberculosis detection in EPTB specimens might vary widely according to specimen type, as it did in another systematic review and meta-analysis of EPTB diagnosis using the Xpert MTB/RIF assay [42]. However, our meta-analysis could not reach a conclusion, partially due to the limited number of studies using the same sample types. Only four studies used CSF to analyze the diagnostic accuracy of LAMP, and there were not enough separate studies using other sample types to carry out meta-analysis. Additionally, these results must be treated with caution, as the heterogeneity between the studies was very significant; this may lead to bias in the results. Further studies using different types of specimens are needed to assess the diagnostic accuracy of LAMP for individual samples.

We observed that incubation time and sample condition in LAMP assays did not affect test results. Different assay types might affect results, e.g., an in-house LAMP assay might be better than the Loopamp MTBC assay. As the heterogeneity between the studies was very significant, further studies using different types of the LAMP assay are needed to assess its sensitivity and specificity.

PCR tests are considered the most effective means of diagnosis [43]. However, these assays, such as Xpert MTB/RIF, are very costly, which is an obstacle to their application in low-income areas. LAMP is gradually being accepted as an alternative test in resource-limited areas due to its relatively small financial burden [35]. In the current meta-analysis, all studies were conducted in low-income countries where medical resources are limited. We observed that the effectiveness of LAMP in EPTB diagnosis was similar to that of Xpert MTB/RIF, which was consistent with a previous study [44]. However, compared with Xpert MTB/RIF, LAMP has shortcomings, such as its inability to determine rifampicin resistance. For low-income areas with low prevalence of drug-resistant tuberculosis, LAMP might be a useful alternative to Xpert MTB/RIF.

Several limitations existed in our review. First, the meta-analysis was limited by the number of studies using different target genes and sample types, particularly those comparing LAMP against culture. Only two target genes and one sample type (CSF) could be analyzed through meta-analysis; the diagnostic validity of the LAMP assay for other target gene and sample types could not be assessed. Some included studies used multiple sample types, which may have led to some bias in the results. Second, the quality of some studies in this analysis was relatively poor. The heterogeneity between the studies was remarkable, and the meta-analysis results should be interpreted with caution.

## Conclusions

In this meta-analysis, we observed that the pooled sensitivity and specificity of LAMP for the detection of EPTB were 77% and 93%, respectively, when compared with a CRS, and 99% and 77%, respectively, when compared with culture. Depending on the assessment of AUC, LAMP showed good diagnostic efficacy. We also found that the diagnostic efficacy of LAMP tests varied according to different target genes; the diagnostic efficacy of MPB64 LAMP was better than that of IS6110 LAMP. The diagnostic accuracy of LAMP for different samples could not be effectively assessed, as the number of studies using different sample types was limited. Additionally, an in-house LAMP assay might be superior to the Loopamp MTBC assay. Because of its low cost, LAMP could be useful in the diagnosis of EPTB, particularly in areas where financial resources are limited and drug-resistant MTB is not prevalent.

## Supporting information

S1 File Search strategy. (DOCX)

S2 File Preferred Reporting Items Systematic Reviews Meta Analyses (PRISMA) 2009 flow diagram. (DOC)

S3 File PRISMA 2009 checklist. (DOC)

S1 Table Characteristics of the included studies. (DOCX)

## References

[1] World Health Organization. Global tuberculosis report. 2016.

[2] Norbis L, Alagna R, Tortoli E, Codecasa LR, Migliori GB, Cirillo DM. Challenges and perspectives in the diagnosis of extrapulmonary tuberculosis. Expert Rev Anti Infect Ther. 2014;12:633–47. doi: 10.1586/14787210.2014.899900 24717112 10.1586/14787210.2014.899900

[3] Aryan E, Makvandi M, Farajzadeh A, Huygen KA, Bifani P, Mousavi SL, et al A novel and more sensitive loop-mediated isothermal amplification assay targeting IS6110 for detection of Mycobacterium tuberculosis complex. Microbiol Res. 2010;165:211–20. doi: 10.1016/j.micres.2009.05.001 19515543 10.1016/j.micres.2009.05.001

[4] Leeflang MM. Systematic reviews and meta-analyses of diagnostic test accuracy. Clin Microbiol Infect. 2014;20:105–13. doi: 10.1111/1469-0691.12474 24274632 10.1111/1469-0691.12474

[5] Moher D, Liberati A, Tetzlaff J, Altman DG. Preferred reporting items for systematic reviews and meta-analyses: the PRISMA statement. PLoS Med 2009;6: e1000097 doi: 10.1371/journal.pmed.1000097 19621072 10.1371/journal.pmed.1000097 PMC2707599

[6] Leeflang MM, Deeks JJ, Gatsonis C, Bossuyt PM. Systematic reviews of diagnostic test accuracy. Ann Intern Med. 2008;149:889–97. 19075208 10.7326/0003-4819-149-12-200812160-00008 PMC2956514

[7] Whiting PF, Rutjes AW, Westwood ME, Mallett S, Deeks JJ, Reitsma JB, et al QUADAS-2: a revised tool for the quality assessment of diagnostic accuracy studies. Ann Intern Med. 2011;155:529–36. doi: 10.7326/0003-4819-155-8-201110180-00009 22007046 10.7326/0003-4819-155-8-201110180-00009

[8] Macaskill P, Gatsonis C, Deeks JJ, Harbord R, Takwoingi Y. Chapter 10: Analysing and Presenting Results. In: Deeks JJ, Bossuyt PM, Gatsonis C, editors. Cochrane Handbook for Systematic Reviews of Diagnostic Test Accuracy Version 1.0, The Cochrane Collaboration, 2010 Available from: http://srdta.cochrane.org/.

[9] Higgins JP, Thompson SG, Deeks JJ, Altman DG. Measuring inconsistency in meta-analyses. BMJ. 2003;327:557–60. doi: 10.1136/bmj.327.7414.557 12958120 10.1136/bmj.327.7414.557 PMC192859

[10] Higgins JP, Thompson SG. Quantifying heterogeneity in a meta-analysis. Stat Med. 2002;21:1539–58. doi: 10.1002/sim.1186 12111919 10.1002/sim.1186

[11] Valenstein PN. Evaluating diagnostic tests with imperfect standards. Am J Clin Pathol. 1990;93:252–8. 2405632 10.1093/ajcp/93.2.252

[12] Reitsma JB, Rutjes AW, Khan KS, Coomarasamy A, Bossuyt PM. A review of solutions for diagnostic accuracy studies with an imperfect or missing reference standard. J Clin Epidemiol. 2009;62:797–806. doi: 10.1016/j.jclinepi.2009.02.005 19447581 10.1016/j.jclinepi.2009.02.005

[13] Schiller I, van Smeden M, Hadgu A, Libman M, Reitsma JB, Dendukuri N. Bias due to composite reference standards in diagnostic accuracy studies. Stat Med. 2016;35:1454–70. doi: 10.1002/sim.6803 26555849 10.1002/sim.6803

[14] Naaktgeboren CA, Bertens LC, van Smeden M, de Groot JA, Moons KG, Reitsma JB. Value of composite reference standards in diagnostic research. BMJ. 2013;347: f5605 doi: 10.1136/bmj.f5605 24162938 10.1136/bmj.f5605

[15] Balne PK, Basu S, Rath S, Barik MR, Sharma S. Loop mediated isothermal amplification assay using hydroxy naphthol blue, conventional polymerase chain reaction and real-time PCR in the diagnosis of intraocular tuberculosis. Indian J Med Microbiol. 2015;33:568–71. doi: 10.4103/0255-0857.167339 26470966 10.4103/0255-0857.167339

[16] Ghosh PK, Chakraborty B, Maiti PK, Ray R. Comparative evaluation of loop-mediated isothermal amplification and conventional methods to diagnose extrapulmonary tuberculosis. Ann Trop Med Pub Health. 2017;10:160–4.

[17] Joon D, Nimesh M, Saluja D. Loop-mediated isothermal amplification as alternative to PCR for the diagnosis of extra-pulmonary tuberculosis. Int J Tuberc Lung Dis. 2015;19:986–91. doi: 10.5588/ijtld.14.0793 26162366 10.5588/ijtld.14.0793

[18] Joon D, Nimesh M, Varma-Basil M, Saluja D. Evaluation of improved IS6110 LAMP assay for diagnosis of pulmonary and extra pulmonary tuberculosis. J Microbiol Methods. 2017;139:87–91. doi: 10.1016/j.mimet.2017.05.007 28514671 10.1016/j.mimet.2017.05.007

[19] Kumar P, Pandya D, Singh N, Behera D. Loop-mediated isothermal amplification assay for rapid and sensitive diagnosis of tuberculosis. J Infect. 2014;69:607–15. doi: 10.1016/j.jinf.2014.08.017 25218428 10.1016/j.jinf.2014.08.017

[20] Modi M, Sharma K, Sharma M, Sharma A, Sharma N, Sharma S, et al Multitargeted loop-mediated isothermal amplification for rapid diagnosis of tuberculous meningitis. Int J Tuberc Lung Dis. 2016;20:625–30. doi: 10.5588/ijtld.15.0741 27084816 10.5588/ijtld.15.0741

[21] Nagdev KJ, Kashyap RS, Parida MM, Kapgate RC, Purohit HJ, Taori GM, et al Loop-mediated isothermal amplification for rapid and reliable diagnosis of tuberculous meningitis. J Clin Microbiol. 2011;49:1861–5. doi: 10.1128/JCM.00824-10 21411583 10.1128/JCM.00824-10 PMC3122663

[22] Sethi S, Dhaliwal L, Dey P, Kaur H, Yadav R, Sethi S. Loop-mediated isothermal amplification assay for detection of Mycobacterium tuberculosis complex in infertile women. Indian J Med Microbiol. 2016;34:322–7. doi: 10.4103/0255-0857.188323 27514954 10.4103/0255-0857.188323

[23] Sharma K, Sharma M, Batra N, Sharma A, Dhillon MS. Diagnostic potential of multi-targeted LAMP (loop-mediated isothermal amplification) for osteoarticular tuberculosis. J Orthop Res. 2017;35:361–5. doi: 10.1002/jor.23293 27175946 10.1002/jor.23293

[24] Sharma M, Sharma K, Sharma A, Gupta N, Rajwanshi A. Loop-mediated isothermal amplification (LAMP) assay for speedy diagnosis of tubercular lymphadenitis: The multi-targeted 60-minute approach. Tuberculosis. 2016;100:114–7. doi: 10.1016/j.tube.2016.07.015 27553418 10.1016/j.tube.2016.07.015

[25] Sun WW, Sun Q, Yan LP, Zhang Q. The application of IS6110-baced loop-mediated isothermal amplification (LAMP) in the early diagnosis of tuberculous meningitis. Oncotarget. 2017;8:57537–42. doi: 10.18632/oncotarget.15734 28915693 10.18632/oncotarget.15734 PMC5593665

[26] Yang B, Wang X, Li H, Li G, Cao Z, Cheng X. Comparison of loop-mediated isothermal amplification and real-time PCR for the diagnosis of tuberculous pleurisy. Lett Appl Microbiol. 2011;53:525–31. doi: 10.1111/j.1472-765X.2011.03141.x 21883320 10.1111/j.1472-765X.2011.03141.x

[27] Liu Y, Guo YL, Jiang GL, Sun Q, Xing AY, Zhang ZD. Application of loop mediated isothermal amplification technique in rapid diagnosis of tuberculous pleurisy. J Clin Pulm Med. 2015;583–5. Chinese.

[28] Zhang SH. Application of laboratory diagnostic methods for urinary tract tuberculosis in urinary tract infection. Nei Mongol J Tradit Chin Med. 2012;31:106–7. Chinese.

[29] Balne PK, Barik MR, Sharma S, Basu S. Development of a loop-mediated isothermal amplification assay targeting the mpb64 gene for diagnosis of intraocular tuberculosis. J Clin Microbiol. 2013;51:3839–40. doi: 10.1128/JCM.01386-13 23966513 10.1128/JCM.01386-13 PMC3889776

[30] Sun WW, Xiao HP. Study on loop mediated isothermal amplification technique for early diagnosis of tuberculous meningitis. Chin J Antituberc. 2016;38:1102–8. Chinese.

[31] Zhou FR, Sun JY, Ma WF. Rapid diagnosis of urinary tuberculosis. Journal of North Pharmacy. 2011;08:22–3. Chinese.

[32] Njiru ZK. Loop-mediated isothermal amplification technology: towards point of care diagnostics. PLoS Negl Trop Dis. 2012;6:e1572 doi: 10.1371/journal.pntd.0001572 22745836 10.1371/journal.pntd.0001572 PMC3383729

[33] Gelaw B, Shiferaw Y, Alemayehu M, Bashaw AA. Comparison of loop-mediated isothermal amplification assay and smear microscopy with culture for the diagnostic accuracy of tuberculosis. BMC Infect Dis. 2017;17:79 doi: 10.1186/s12879-016-2140-8 28095790 10.1186/s12879-016-2140-8 PMC5240421

[34] Nliwasa M, MacPherson P, Chisala P, Kamdolozi M, Khundi M, Kaswaswa K, et al The Sensitivity and Specificity of Loop-Mediated Isothermal Amplification (LAMP) Assay for Tuberculosis Diagnosis in Adults with Chronic Cough in Malawi. PLoS One. 2016;11:e0155101 doi: 10.1371/journal.pone.0155101 27171380 10.1371/journal.pone.0155101 PMC4865214

[35] Nagai K, Horita N, Yamamoto M, Tsukahara T, Nagakura H, Tashiro K, et al Diagnostic test accuracy of loop-mediated isothermal amplification assay for Mycobacterium tuberculosis: systematic review and meta-analysis. Sci Rep. 2016;6:39090 doi: 10.1038/srep39090 27958360 10.1038/srep39090 PMC5153623

[36] Park K, Kim J, Lee J, Hwang Y, Jeon K, Koh W, et al Comparison of the Xpert MTB/RIF and Cobas TaqMan MTB assays for detection of Mycobacterium tuberculosis in respiratory specimens. J Clin Microbiol. 2013;51:3225–7. doi: 10.1128/JCM.01335-13 23863563 10.1128/JCM.01335-13 PMC3811628

[37] Steingart K, Schiller I, Horne D, Pai M, Boehme C, Dendukuri N. Xpert® MTB/RIF assay for pulmonary tuberculosis and rifampicin resistance in adults. Cochrane Database Syst Rev. 2014:CD009593 doi: 10.1002/14651858.CD009593.pub3 24448973 10.1002/14651858.CD009593.pub3 PMC4470349

[38] Nagdev KJ, Kashyap RS, Parida MM, Kapgate RC, Purohit HJ, Taori GM, et al Loop-mediated isothermal amplification for rapid and reliable diagnosis of tuberculous meningitis. J Clin Microbiol. 2011;49:1861–5. doi: 10.1128/JCM.00824-10 21411583 10.1128/JCM.00824-10 PMC3122663

[39] Vadwai V, Shetty A, Rodrigues C. Using likelihood ratios to estimate diagnostic accuracy of a novel multiplex nested PCR in extra-pulmonary tuberculosis. Int J Tuberc Lung Dis. 2012;16:240–7. doi: 10.5588/ijtld.11.0322 22236927 10.5588/ijtld.11.0322

[40] Raj A, Singh N, Gupta KB, Chaudhary D, Yadav A, Chaudhary A, et al Comparative Evaluation of Several Gene Targets for Designing a Multiplex-PCR for an Early Diagnosis of Extrapulmonary Tuberculosis. Yonsei Med J. 2016;57:88–96. doi: 10.3349/ymj.2016.57.1.88 26632387 10.3349/ymj.2016.57.1.88 PMC4696977

[41] Sharma K, Sinha SK, Sharma A, Nada R, Prasad KK, Goyal K, et al Multiplex PCR for rapid diagnosis of gastrointestinal tuberculosis. J Global Infect Dis. 2013;5:49–53. 10.4103/0974-777X.112272 PMC3703210 23853431

[42] Denkinger C, Schumacher S, Boehme C, Dendukuri N, Pai M, Steingart K. Xpert MTB/RIF assay for the diagnosis of extrapulmonary tuberculosis: a systematic review and meta-analysis. Eur Respir J. 2014;44:435–46. doi: 10.1183/09031936.00007814 24696113 10.1183/09031936.00007814

[43] World Health Organization. The use of a commercial loop-mediated isothermal amplification assay (TB-lamp) for the detection of tuberculosis: expert group meeting report, Geneva: May 2013.

[44] Penz E, Boffa J, Roberts DJ, Fisher D, Cooper R, Ronksley PE, et al Diagnostic accuracy of the Xpert(R) MTB/RIF assay for extra-pulmonary tuberculosis: a meta-analysis. Int J Tuberc Lung Dis. 2015;19:278–84. doi: 10.5588/ijtld.14.0262 25686134 10.5588/ijtld.14.0262

