# Supplementary material for: Diagnostic accuracy of the loop-mediated isothermal amplification assay for extrapulmonary tuberculosis: A meta-analysis
Source: PLoS One. 2018 Jun 26;13(6):e0199290. doi: 10.1371/journal.pone.0199290 (PMC6019099; doi:10.1371/journal.pone.0199290)
Supplement: S1 File — (DOCX) [file pone.0199290.s001.docx]

Pubmed and The Cochrane Library

#1: “Loop-Mediated Isothermal Amplification” OR LAMP

#2: "Tuberculosis"[Mesh] OR Tuberculoses OR “Kochs Disease” OR “Disease, Kochs” OR “Koch's Disease” OR “Disease, Koch's” OR “Koch Disease” OR TB

#3: #1 AND #2

Cochrane Library: 11 articles

Pubmed: 264 articles

Embase

#1: 'Loop-Mediated Isothermal Amplification' OR LAMP

#2: 'tuberculosis'/exp OR Tuberculoses OR 'Kochs Disease' OR 'Disease, Kochs' OR 'Koch's Disease' OR 'Disease, Koch's' OR 'Koch Disease' OR TB

#3: #1 AND #2

Embase: 328 articles

CNKI and Wangfang Database

#1: 环介导等温核酸扩增技术OR LAMP

#2: 结核

#3: #1 AND #2

Wanfang: 98 articles

CNKI: 99 articles
