# Supplementary material for: Diagnostic accuracy of the loop-mediated isothermal amplification assay for extrapulmonary tuberculosis: A meta-analysis
Source: PLoS One. 2018 Jun 26;13(6):e0199290. doi: 10.1371/journal.pone.0199290 (PMC6019099; doi:10.1371/journal.pone.0199290)
Supplement: S2 File — (DOC) [file pone.0199290.s002.doc]

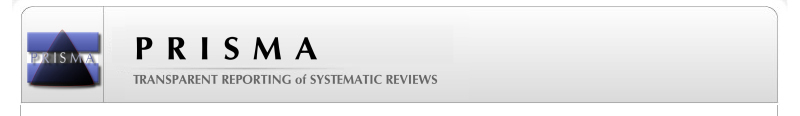
**PRISMA 2009 Flow Diagram**

**Screening**

**Included**

**Eligibility**

**Identification**

Records identified through database searching
(n = 800)

Additional records identified through other sources
(n=0)

Records after duplicates removed
(n=208)

Records screened
(n=592)

Records excluded
(n =539 )

Full-text articles assessed for eligibility
(n=53)

Full-text articles excluded, with reasons (n=39)

Review (n=4）

Non clinical specimen (n=8)

Unspecified gold standard (n=3)

Only abstract (n=3)

Data duplication (n=3)

Not human studies (n=4)

Not about LAMP (n=8)

Not sensitivity (n=6)

Records included in qualitative synthesis
(n=14)

Records included in quantitative synthesis (meta-analysis)
(n=14)
