# Supplementary material for: Diagnostic accuracy of the loop-mediated isothermal amplification assay for extrapulmonary tuberculosis: A meta-analysis
Source: PLoS One. 2018 Jun 26;13(6):e0199290. doi: 10.1371/journal.pone.0199290 (PMC6019099; doi:10.1371/journal.pone.0199290)
Supplement: S1 Table — (DOCX) [file pone.0199290.s004.docx]

| Author | Year | Country | Language | TP(n) | FP(n) | FN(n) | TN(n) | Reference | Gene | Sample Type | HIV |
| --- | --- | --- | --- | --- | --- | --- | --- | --- | --- | --- | --- |
| BinFeng Yang_a_ | 2011 | China | English | 18 | 0 | 54 | 24 | CRS | IS1081 | PE | Negative |
| BinFeng Yang_b_ | 2011 | China | English | 35 | 4 | 37 | 20 | CRS | IS1081 | PE | Negative |
| Nagdev, K. J. | 2011 | India | English | 15 | 2 | 2 | 8 | CRS | IS6110 | CSF | Unknown |
| Shihui Zhang | 2012 | China | Chinese | 31 | 6 | 2 | 19 | Culture | Unknown | Urine | Unknown |
| Kumar, P. | 2014 | India | English | 22 | 23 | 0 | 32 | Culture | esat6 | Extrapulmonary samples | Unknown |
| Yang Liu | 2015 | China | Chinese | 44 | 2 | 14 | 30 | CRS | hspX | PE | Unknown |
| Joon, D._a_ | 2015 | India | English | 28 | 23 | 2 | 262 | Culture | sdaA | Extrapulmonary samples | Unknown |
| Joon, D._b_ | 2015 | India | English | 49 | 2 | 4 | 260 | CRS | sdaA | Extrapulmonary samples | Unknown |
| Balne, P. K. | 2015 | India | English | 25 | 0 | 8 | 20 | CRS | MPB64 | Vitreous and aqueous humor | Unknown |
| Sharma, M._a_ | 2016 | India | English | 96 | 0 | 24 | 50 | CRS | IS6110 | FNA | Unknown |
| Sharma, M._b_ | 2016 | India | English | 103 | 0 | 17 | 50 | CRS | MPB64 | FNA | Unknown |
| Sethi, S._a_ | 2016 | India | English | 5 | 57 | 2 | 236 | Culture | IS6110 | Endometrial biopsy samples | Unknown |
| Sethi, S._b_ | 2016 | India | English | 45 | 17 | 23 | 215 | CRS | IS6110 | Endometrial biopsy samples | Unknown |
| Modi, M._a_ | 2016 | India | English | 46 | 78 | 4 | 122 | Culture | IS6110 | CSF | Unknown |
| Modi, M._b_ | 2016 | India | English | 48 | 82 | 2 | 118 | Culture | MPB64 | CSF | Unknown |
| Modi, M._c_ | 2016 | India | English | 124 | 0 | 26 | 100 | CRS | IS6110 | CSF | Unknown |
| Modi, M._d_ | 2016 | India | English | 130 | 0 | 20 | 100 | CRS | MPB64 | CSF | Unknown |
| Wenwen Sun_a_ | 2017 | China | English | 74 | 2 | 98 | 26 | CRS | IS6110 | CSF | Unknown |
| Wenwen Sun_b_ | 2017 | China | English | 20 | 56 | 2 | 122 | Culture | IS6110 | CSF | Unknown |
| Sharma, K._a_ | 2017 | India | English | 75 | 0 | 15 | 50 | CRS | IS6110 | Synovial fluid and pus | Unknown |
| Sharma, K._b_ | 2017 | India | English | 79 | 0 | 11 | 50 | CRS | MPB64 | Synovial fluid and pus | Unknown |
| Joon, D._a_ | 2017 | India | English | 43 | 0 | 10 | 262 | CRS | IS6110 | Extrapulmonary samples | Unknown |
| Joon, D._b_ | 2017 | India | English | 26 | 17 | 4 | 268 | Culture | IS6110 | Extrapulmonary samples | Unknown |
| Ghosh, P. K. | 2017 | India | English | 22 | 1 | 1 | 21 | CRS | Unknown | Extrapulmonary samples | Unknown |

Characteristics of the included studies. CRS: Composite reference standard. PE: Pleural effusion. FNA: Fine needle aspiration. CSF: Cerebrospinal fluid. Extrapulmonary samples included PE, CSF, and others.
